# Supplementary material for: Heat adaptation of phage T7 under an extended genetic code
Source: Virus Evol. 2021 Dec 1;7(2):veab100. doi: 10.1093/ve/veab100 (PMC8923235; doi:10.1093/ve/veab100)
Supplement: veab100_Supp [file veab100_supp.zip › Sequencing_Analysis.zip!/Sequencing_Analysis/A2/A2_52_S18_L001_R1_001_fastqc.html]

A2\_52\_S18\_L001\_R1\_001.fastq FastQC Report 

FastQC Report

Mon 7 Aug 2017  
A2\_52\_S18\_L001\_R1\_001.fastq

## Summary

- Basic Statistics
- Per base sequence quality
- Per tile sequence quality
- Per sequence quality scores
- Per base sequence content
- Per sequence GC content
- Per base N content
- Sequence Length Distribution
- Sequence Duplication Levels
- Overrepresented sequences
- Adapter Content
- Kmer Content

## Basic Statistics

| Measure | Value |
| --- | --- |
| Filename | A2\_52\_S18\_L001\_R1\_001.fastq |
| File type | Conventional base calls |
| Encoding | Sanger / Illumina 1.9 |
| Total Sequences | 825889 |
| Sequences flagged as poor quality | 0 |
| Sequence length | 301 |
| %GC | 44 |

## Per base sequence quality

## Per tile sequence quality

## Per sequence quality scores

## Per base sequence content

## Per sequence GC content

## Per base N content

## Sequence Length Distribution

## Sequence Duplication Levels

## Overrepresented sequences

No overrepresented sequences

## Adapter Content

## Kmer Content

| Sequence | Count | PValue | Obs/Exp Max | Max Obs/Exp Position |
| --- | --- | --- | --- | --- |
| GCCTTTT | 690 | 0.0 | 151.77536 | 295 |
| GGGCGTT | 185 | 2.2224413E-8 | 55.81081 | 9 |
| GCATTTT | 770 | 0.0 | 55.551952 | 295 |
| TCCCCGG | 300 | 0.0 | 55.066666 | 270-274 |
| CCCGGGC | 190 | 0.0 | 54.34211 | 270-274 |
| GACTTTT | 490 | 0.0 | 54.183674 | 295 |
| TGATCCT | 355 | 0.0 | 54.014084 | 295 |
| TCCTTTT | 1850 | 0.0 | 53.41892 | 295 |
| TGGCGCC | 85 | 0.0071872193 | 52.058826 | 9 |
| CCCCGGG | 210 | 0.0 | 51.97619 | 270-274 |
| CGGGCTC | 205 | 0.0 | 51.804874 | 275-279 |
| GCCTGTG | 90 | 0.009009074 | 49.166664 | 295 |
| GGGCTCA | 250 | 0.0 | 46.02 | 275-279 |
| CGTGCGC | 290 | 0.0 | 45.77586 | 235-239 |
| CAGGCGG | 265 | 0.0 | 44.528305 | 240-244 |
| CCCCGGC | 100 | 0.0 | 44.25 | 270-274 |
| AACCTGG | 320 | 0.0 | 44.25 | 280-284 |
| GCCTGTT | 235 | 1.4620127E-7 | 43.93617 | 295 |
| GGGTTTA | 170 | 0.0 | 43.382355 | 220-224 |
| CCGGGCT | 225 | 0.0 | 43.26667 | 275-279 |

Produced by FastQC (version 0.11.3)
